# The complete chloroplast genome of *Bupleurum longicaule* var. *strictum*, an annual herb endemic to China

**DOI:** 10.1080/23802359.2020.1718024

**Published:** 2020-01-27

**Authors:** Lucun Yang, Feng Xiong, Yuanming Xiao, Jingjing Li, Chen Chen, Guoying Zhou

**Affiliations:** a Northwest Institute of Plateau Biology, Chinese Academy of Sciences, Xining, China;; b Qinghai Key Laboratory of Qinghai-Tibet Plateau Biological Resources, Xining, China;; c Research Center for Biological Resources in Qinghai-Tibet Plateau, University of Chinese Academy of Sciences, Beijing, China;; d College of Life Science, Qinghai Normal University, Xining, China

**Keywords:** *Bupleurum*, *Bupleurum longicaule* var. *Strictum*, Chloroplast genome

## Abstract

*Bupleurum* species are well-known for their antipyretic, analgesic, gallbladder and other functions so that they are very popular in traditional Chinese medicine. However, to our knowledge, there is no completed chloroplast genome of *Bupleurum* genus in China. In the present study, we determined the complete chloroplast genome sequences for *Bupleurum longicaule* var. *strictum* using IIumina sequencing. *Bupleurum longicaule* var. *strictum* is 155,578 bp which is composed of two inverted repeats (IR: 26,257bp), a large single copy region (LSC: 86,977 bp), and a small single-copy region (SSC: 16,087 bp). The overall AT content is 65.32%. The chloroplast genome includes a total of 51 functional genes including 15 protein-coding genes and 36 tRNA. A total of 10 genes were duplicated in the IR regions including seven tRNA and three protein-coding genes. Phylogenetic analysis suggested that *B. longicaule* var. *strictum* formed a monophyletic clade.

The genus *Bupleurum*, belonging to the Umbelliferae family, encompasses more than 180 species distributed mainly in the Northern Hemisphere. In China, the genus consists of 42 species and 17 varieties, 22 of which are recognized as endemisms (Shan and She 1979). *Bupleurum* species are well-known for their antipyretic, analgesic, gallbladder, and other functions so that they are very popular in traditional Chinese medicine (Luo and Jin 1991; Pan 2006; Tan et al. 2007). However, to our knowledge, there is no completed chloroplast genome of *Bupleurum* genus in China. In the present study, we report the completed chloroplast genome of *Bupleurum longicaule* var. *strictum* based on the next-generation sequencing method. The chloroplast genome will contribute to molecular phylogeny, genetic information, mechanism of evolution or adoption.

A wild individual of *B. longicaule* var. *strictum* was collected from Xinping village (101°37.556′E, 36°34.460′N, 2510 m), Huangzhong country in Qinghai province of China (Voucher specimen: QHGC20190823, HNWP). The Illumina sequencing process was as follows: extraction of Genome DNA, fragmentation of DNA, construction of Illumina pair-end library and sequencing with the Illumina HiSeq 4000 platform. After filtering reads with low-sequencing quality, high-quality clean data were assembled with the SPAdes (Bankevich et al. 2012). And then, annotation was performed with DOGMA (Wyman et al. 2004). The annotated genomic sequence has been submitted to GenBank with the accession number SRR10579389.

The total length of the complete chloroplast genome of *B*. *longicaule* var. *strictum* is 155,578 bp which is composed of two inverted repeats (IR: 26,257bp), a large single-copy region (LSC: 86,977 bp) and a small single-copy region (SSC: 16,087 bp). The overall AT content is 65.32%. The chloroplast genome includes a total of 51 functional genes including 15 protein-coding genes and 36 tRNA. A total of 10 genes were duplicated in the IR regions including seven tRNA and three protein-coding genes. The genome organization, gene/intron content and gene relative positions of the newly sequenced plastid genome were almost identical to other Umbelliferae species.

The phylogenetic tree was constructed with the complete chloroplast genomes of *B. longicaule* var. *strictum* and 46 other species from Umbelliferae, and *Panax stipuleanatus* was used as an outgroup. The result showed that *B. longicaule* var. *strictum* formed a monophyletic clade (Figure 1).

**Figure 1.**
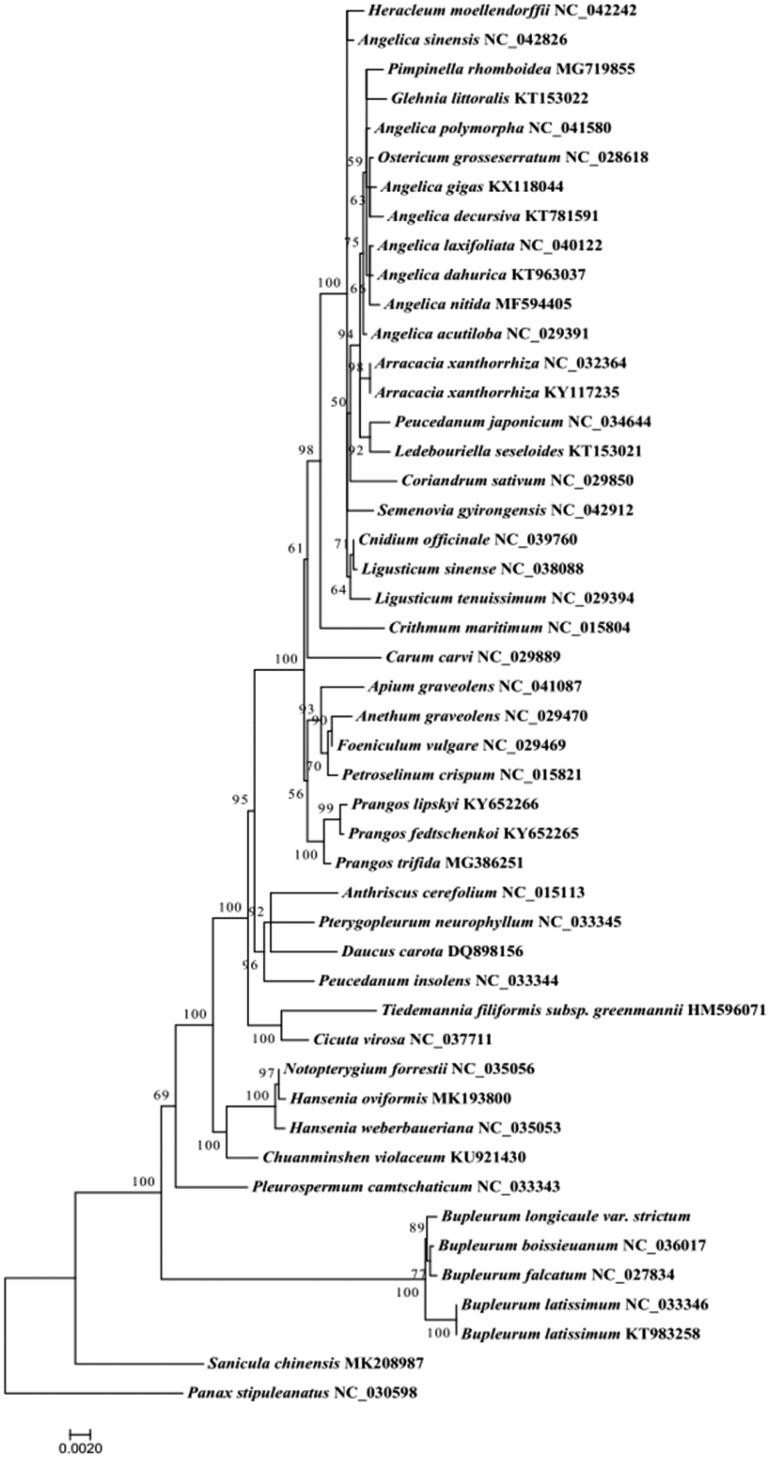
Maximum likelihood phylogenetic tree based on 48 complete chloroplast genome sequences.
